# Immobilization of an Iridium Pincer Complex in a Microporous Polymer for Application in Room‐Temperature Gas Phase Catalysis

**DOI:** 10.1002/anie.202004092

**Published:** 2020-08-31

**Authors:** Michaela König, Massimo Rigo, Nicolas Chaoui, Trung Tran Ngoc, Jan Dirk Epping, Johannes Schmidt, Pradip Pachfule, Meng‐Yang Ye, Matthias Trunk, Johannes F. Teichert, Matthias Drieß, Arne Thomas

**Affiliations:** ^1^ Institut für Chemie Technische Universität Berlin Hardenbergstrasse 40 10623 Berlin Germany; ^2^ Institut für Chemie Technische Universität Berlin Straße des 17. Juni 115 10623 Berlin Germany

**Keywords:** catalysis, gas phase, hydrogenation, microporous polymer network, pincer

## Abstract

An iridium dihydride pincer complex [IrH_2_(POCOP)] is immobilized in a hydroxy‐functionalized microporous polymer network using the concepts of surface organometallic chemistry. The introduction of this novel, truly innocent support with remote OH‐groups enables the formation of isolated active metal sites embedded in a chemically robust and highly inert environment. The catalyst maintained high porosity and without prior activation exhibited efficacy in the gas phase hydrogenation of ethene and propene at room temperature and low pressure. The catalyst can be recycled for at least four times.

## Graphical abstract

An iridium pincer complex, immobilized in a microporous polymer network, has been used as an efficient and recyclable catalyst for the gas phase hydrogenation of ethene and propene at room temperature and low pressure.

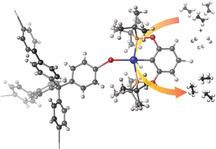

Organometallic pincer complexes have received tremendous attention due to their great number of applications in homogeneous catalysis, such as dehydrogenation, coupling, hydrogen transfer, as well as aldol and Michael reactions.[^1^, ^2^, ^3^, ^4^, ^5^, ^6^, ^7^, ^8^, ^9^]

Immobilization of pincer complexes by covalent bond formation with the supporting material, such as silica and metal oxides[^10^, ^11^, ^12^, ^13^, ^14^, ^15^, ^16^, ^17^, ^18^] as well as metal‐organic frameworks[^19^, ^20^, ^21^, ^22^, ^23^] have been reported. Immobilization of molecular catalysts onto porous materials combines advantages of homogeneous as well as heterogeneous catalysis, such as high conversion rates and good reusability.[^24^, ^25^] Most importantly, molecular catalysts can be made accessible for gas phase catalytic reactions using this approach. Porous silica is probably the most common support used for immobilization of molecular catalysts and surface organometallic chemistry; however, this inorganic support provides many, densely packed hydroxy functionalities, so that pre‐treatments are essential to control the binding of a metal organic complex either on one, two or even more hydroxy groups. Indeed various analytical tools are needed to elucidate the binding state of organometallic complexes on silica supports.[^24^, ^25^]

Microporous polymer networks (MPNs) exhibit some intriguing properties such as the accessible high surface area, chemical robustness, and tunable functionality, which make these materials interesting for various applications and also for immobilization of molecular catalysts.^[26]^ Even though MPNs with various functional moieties in their backbone have been presented, some examples are also solely composed of carbon and hydrogen and show no extended π‐conjugation, such as the highly porous PAF‐1^[27]^ or PPN‐6.^[28]^ If organometallic species could be grafted on such a MPN, this would ensure homogeneous distribution of a single‐site catalyst over the entire surface area of a non‐functional and inert organic polymer, thus a truly “innocent” support. Such kind of materials have been reported to be active in catalysis application in condensed phase,[^29^, ^30^, ^31^, ^32^, ^33^, ^34^, ^35^, ^36^, ^37^, ^38^, ^39^, ^40^, ^41^, ^42^, ^43^, ^44^, ^45^, ^46^, ^47^, ^48^, ^49^] but rarely in gas phase.^[50]^

Herein, we present the immobilization of an organometallic molecular iridium pincer complex on a high‐surface‐area microporous polymer network (Scheme 1) and its application in catalytic gas phase hydrogenation of ethene and propene. The novel MPN features an inert environment with isolated anchor points for the immobilization of the highly active metal‐organic complex, ensuring the formation of a single‐site catalyst.

**Scheme 1:**
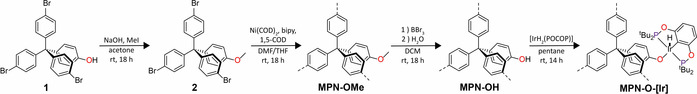
Synthesis of methoxytetraphenylmethane Polymer **MPN‐OMe**, post‐synthetic modification towards hydroxy functionalized **MPN‐OH** and immobilization of iridium pincer complex [IrH_2_(POCOP)] onto **MPN‐OH** yielding **MPN‐O‐[Ir]**.

In order to provide a reactive hydroxy functionality as anchor point while also following the tetraphenylmethane structural motif of the highly porous PAF‐1^[27]^/PPN‐6,^[28]^ tris(4‐bromophenyl)‐methanol^[51]^ was synthesized and converted into 4‐hydroxyphenyl‐tris(4‐bromophenyl)methane **1**. To avoid possible interactions with the utilized metal species during polymerization, the hydroxy group was protected by methylation to yield 4‐methoxyphenyl‐tris(4‐bromophenyl)methane **2** (Scheme 1, further details can be found in the supporting information). The structures of **1** and **2** were confirmed by single crystal X‐ray diffraction analyses (SI Figure S1–S3). Since its first application in 2009^[52]^ the nickel‐mediated Yamamoto polymerization has been a convenient method for the synthesis of microporous polymer networks. Following this methodology, **2** was successfully converted into **MPN‐OMe** with a *SA*_BET_ of 1096 m^2^ g^−1^. The assumed chemical structure was confirmed by ^13^C CP/MAS‐NMR spectroscopy (Figure 1 a). The signals of the quaternary methane carbon atom and of the methoxy group could be identified in the aliphatic region at 60 ppm and 50 ppm, respectively. For the aromatic carbon atoms several signals between 155 and 109 ppm were found, whereby the signal at 155 ppm can be most probably assigned to the C_Ar_‐O. To recover the hydroxy group the methylated polymer **MPN‐OMe** was quantitatively deprotected by successive treatment with BBr_3_ and H_2_O to yield a microporous polymer **MPN‐OH** with a *SA*_BET_ of 1087 m^2^ g^−1^. No structural collapse after post‐modification is indicated by similar type I isotherms (Figure 3) and *SA*_BET_. The successful demethylation was confirmed by ^13^C CP/MAS‐NMR spectroscopy (Figure 1) due to the absence of the methoxy signal at 50 ppm. A high‐field shift of the C_Ar_‐O signal from 155 to 151 ppm and a shift of the signal at 109 ppm to 111 ppm is observed after demethylation. The absence of boron species after hydrolysis was confirmed by ^11^B MAS‐NMR spectroscopy of **MPN‐OH** (Figure S5). **MPN‐OMe** and **MPN‐OH** were analyzed by thermogravimetric analysis and showed no weight loss until 400 °C in air (Figure S6). Control experiments proved that the protection‐postsynthetic deprotection route yields a material far superior to the product obtained by direct Yamamoto polymerization of **1** (Scheme S2). It stands to reason that due to interference of the free hydroxy groups with the highly sensitive employed Ni^0^ species a strong decrease of yield and surface area is found (Figure S7).

**Figure 1:**
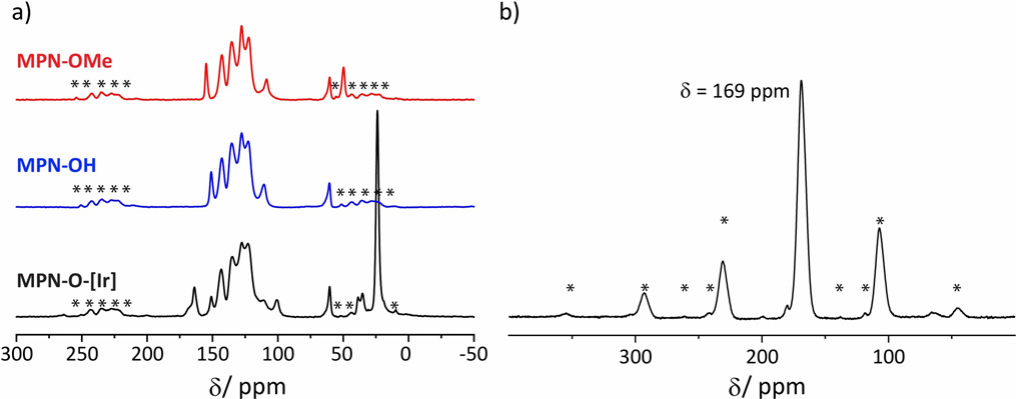
a) Demethylation and immobilization of Ir pincer complex monitored by ^13^C CP/MAS‐NMR spectroscopy: **MPN‐OMe** (red), **MPN‐OH** (blue), and immobilized Ir pincer complex **MPN‐O‐[Ir]** (black). b) ^31^P MAS‐NMR spectroscopy of immobilized iridium pincer complex **MPN‐O‐[Ir]**. Asterisks denote spinning sidebands.

The tridentate iridium pincer complex [IrH_2_(POCOP)] was synthesized according to literature.^[53]^ Then, a pentane solution of [IrH_2_(POCOP)] was added to a suspension of **MPN‐OH** in pentane at room temperature under inert conditions. While stirring overnight the polymer swelled and its color turned from beige to orange (Figure 3 b). Filtration and drying in vacuum yielded the immobilized iridium pincer complex **MPN‐O‐[Ir]** (Scheme 1). To explore the binding geometry of the immobilized pincer complex a model compound was synthesized and analyzed by single crystal *x*‐ray diffraction analysis (Figure S4).

Successful immobilization of the iridium pincer complex was confirmed by solid state NMR spectroscopy (Figure 1). In the ^13^C CP/MAS‐NMR spectrum additional peaks in the aromatic region at 167, 164 and 101 ppm are observed due to the phosphinoxyphenyl ligand of the pincer complex. The signal at 24 ppm can be assigned to the *tert*‐butyl methyl groups, and both signals at 39 and 35 ppm can be assigned to the quaternary *tert*‐butyl carbon atoms. Due to non‐identical chemical environments inside a solid, two signals instead of one are observed for the quaternary *tert*‐butyl carbon atoms. The ^31^P MAS‐NMR spectrum confirms the intact pincer ligand as its chemical shift of 169 ppm closely resembles the values reported in literature.^[11]^ Minute signals at 200 and 180 ppm can be ascribed to traces of uncomplexed **[IrH_2_(POCOP)]** and **[IrHCl(POCOP)]** species. The signal at 64 ppm occurs most likely due to decomposed iridum catalyst and can be assumed to be di‐*tert*‐butyl‐phosphine oxide.^[54]^

The elemental composition of **MPN‐O‐[Ir]** was explored by X‐ray photoelectron spectroscopy (XPS), revealing a 1:2 ratio of iridium to phosphorus which confirms again the immobilization of the intact iridium pincer complex. The high‐resolution C 1s core‐level spectrum of **MPN‐O‐[Ir]** gives two peaks at 284.8 and 286.1 eV, which are attributed to C−C and C−O species, respectively. Two peaks in the O 1s spectrum at 530.1 and 533.2 eV can be assigned to a metal oxygen species, in this case Ir‐O, and an organic oxygen species, respectively. The P 2p spectrum shows a doublet at 131.8 eV, which can be assigned to a P‐O species and a doublet at 133.2 eV which most probably corresponds to di‐tert‐butyl‐phosphine oxide. This confirms the findings in the ^31^P solid state NMR analysis. A doublet signal at 61.9 and 65.0 eV in the Ir 4f core‐level spectrum indicates the presence of one iridium species. The iridium content was determined by inductively coupled plasma optical emission spectroscopy (ICP‐OES) with a value of 15.7 wt %, which is close to the theoretical value of 20.8 wt %, indicating a functionalization degree of 75.5 %. A spherical morphology often observed for microporous polymers is seen by scanning electron microscopy (SEM). Elemental mapping furthermore shows the homogeneous distribution of iridium and phosphorus within **MPN‐O‐[Ir]** (Figure 2 b).

**Figure 2:**
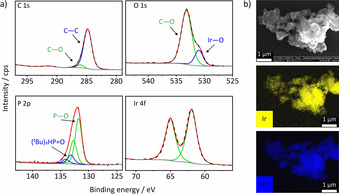
a) C 1s, O 1s, P 2p, and Ir 4f XPS spectra of immobilized iridium pincer complex **MPN‐O‐[Ir]**. Dashed line belongs to the measured spectra, red line belongs to the fitted spectra, green and blue lines belong to the fitted signals. b) Scanning electron microscopy and elemental mapping of **MPN‐O‐[Ir]**. Yellow belongs to iridium, blue belongs to phosphorus.

Porosity was evaluated for **MPN‐O‐[Ir]** by low‐pressure argon sorption studies and compared to **MPN‐OMe** and **MPN‐OH**. All samples showed high gas uptake at low pressures, which is characteristic for microporous materials (Figure 3 a). Using the Brunauer–Emmett–Teller (BET) model, surface areas (*SA*_BET_) were determined to be 1096, 1087 and 451 m^2^ g^−1^ for **MPN‐OMe**, **MPN‐OH** and **MPN‐O‐[Ir]**, respectively. After immobilization of the iridium catalyst, the *SA*_BET_ decreased from 1087 to 451 m^2^ g^−1^, which is almost the expected *SA*_BET_ due to the large increase in molecular weight by a factor of 2.8, assuming full conversion of hydroxy groups. The pore size distributions were calculated from quenched solid density functional theory (QSDFT) and resulted in 0.69 nm for **MPN‐OMe** and **MPN‐OH** and 0.56 nm for **MPN‐O‐[Ir]** (Figure S8).

**Figure 3:**
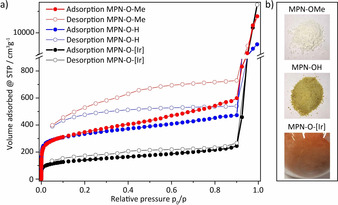
a) Ar sorption analysis at 87 K of **MPN‐OMe** (red), **MPN‐OH** (blue), and **MPN‐O‐[Ir]** (black). b) Optical images of **MPN‐OMe, MPN‐OH**, and **MPN‐O‐[Ir]**.

The immobilized iridium pincer complex was investigated in the catalytic hydrogenation of ethene and propene. There have been reports wherein different complexes immobilized on various supports were studied for gas phase hydrogenation of alkenes (Table S1).[^11^, ^20^, ^50^, ^55^, ^56^, ^57^] But, while immobilized metal catalysts in MPNs have been frequently used as catalyst in liquid phase, as mentioned before, functionalized MPNs are rarely applied for a gas phase catalytic reaction.^[50]^ The permanent porosity of MPNs however is a feature, which is not necessarily needed in solution‐based reactions as any non‐porous but swellable crosslinked polymer could be equally efficient. Indeed, stable permanent surface areas and porosities of MPNs could be much better exploited and will yield a significant benefit when applied in gas phase catalytic reactions.

In this case, the catalytic hydrogenation of alkene and formation of alkane was monitored by ^1^H NMR gas phase spectroscopy. **MPN‐O‐[Ir]** was transferred to an intermediate pressure NMR tube (2.5 mL) which was subsequently evacuated, followed by addition of a 1:1 mixture of alkene and hydrogen (2 bar). After the addition the NMR tube was immediately inserted into the NMR spectrometer and ^1^H gas phase NMR spectra were collected. The obtained signals for alkene and alkane were normalized, integrated, converted into mole fraction and plotted as a function of time (Figure 4 a and b). The hydrogenation reactions of ethene and propene were accomplished after 2 h almost quantitatively. Comparing the conversions of the hydrogenation of ethene and propene (Figure 4 c and Table S2) it is noticeable that the formation of ethane is slightly faster. This can be explained by the higher reactivity of ethene in comparison to propene but may additionally result from the smaller kinetic diameter of ethene and therefore easier access and diffusion through the micropores of the catalytic system.

**Figure 4:**
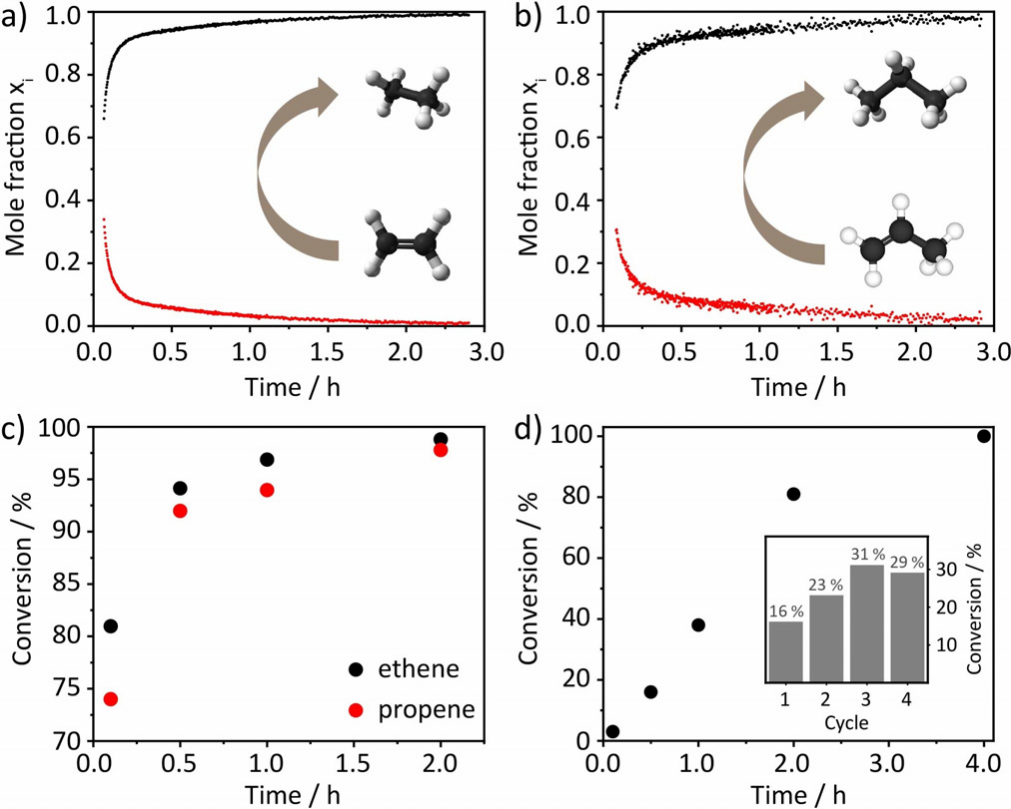
a) Catalytic hydrogenation of ethene monitored by ^1^H gas phase NMR spectroscopy in a 2.5 mL reactor. Reaction conditions: 3 mg catalyst **MPN‐O‐[Ir]**, no solvent, room temperature, 1:1 ratio of ethene to hydrogen, initial total pressure of 2 bar. b) Catalytic hydrogenation of propene monitored by ^1^H gas phase NMR spectroscopy in a 2.5 mL reactor. Reaction conditions: 3 mg catalyst **MPN‐O‐[Ir]**, no solvent, room temperature, 1:1 ratio of propene to hydrogen, initial total pressure of 2 bar. c) Comparison of the hydrogenation of ethene and propene in a 2.5 mL reactor. d) Catalytic hydrogenation of ethene in a 55 mL reactor. Reaction conditions: 8 mg catalyst **MPN‐O‐[Ir]**, no solvent, room temperature, 1:1 ratio of ethene to hydrogen, initial total pressure of 2 bar. Inset image shows the recyclability of **MPN‐O‐[Ir]** for four cycles.

Owing to the rapidity of the reaction in the NMR tube, which causes difficulties in following the reaction, the hydrogenation of ethene was further conducted on a larger scale to evaluate kinetic data in a more reliable fashion as this allows reducing the ratio of catalyst per substrates (Table 1). The reaction follows a seemingly linear pathway for the first two hours and is complete after 4 hours at room temperature (Figure 4 d).

**Table 1:** Catalytic hydrogenation of ethene at room temperature.^[a]^

Entry	*t* [h]	Cycle	Conversion [%]	TON	TOF [min^−1^]
1	0.1	–	3	11	1.9
2	0.5	I	16	56	1.9
3	0.5	II	23	84	2.8
4	0.5	III	31	111	3.7
5	0.5	IV	29	105	3.5
6	1	–	38	135	2.3
7	2	–	81	290	2.4
8	4	–	100	360	–

[a] Reaction conditions: 8 mg catalyst **MPN‐O‐[Ir]**, no solvent, room temperature, 1:1 ratio of ethene to hydrogen, initial total pressure of 2 bar, reactor volume 55 mL. The conversion was determined by integration of the ^1^H NMR gas phase spectra.

After 30 min the conversion to ethane was 16 % which equals to a TON of 56 and a TOF of 1.9 min^−1^. In comparison to a pincer complex supported on mesoporous silica (SBA‐15)^[11]^ the MPN supported catalyst shows a much higher activity per catalyst mass but a lower one when compared to the number of active, that is, Ir sites (see Table S1). One reason can be the diffusion limitation due to the smaller pores. Apart from that it is conceivable that some active sites inside the material are not accessible. It should be noted that a possible heating effect of the catalyst due to the exothermic hydrogenation reaction could not be detected on the polymeric powder catalyst, but has to be also considered when discussing the reaction kinetics.

The catalyst **MPN‐O‐[Ir]** could be reused for at least four times after evacuation and removal of the product under vacuum for 15 min (Inset Figure 4 d). All recycle runs where performed with a reaction time of 30 min as to reach not full conversion. Surprisingly, the conversion increased from the first to the third cycle from 16 % to 31 %. It cannot be excluded that some inactive iridium sites are formed during catalyst preparation, for example, by contact with some traces of air or moisture. During the hydrogenation, that is, under H_2_ atmosphere, such inactive sites are probably regenerated to the iridium‐hydrido‐complex, which is described as the actual catalyst for the hydrogenation of ethene.^[18]^ This will lead to an increase in activity when the catalyst is recycled. After the complex is formed it remains as such in the subsequent runs and is immediately active. The decrease of the conversion in the fourth cycle is within margin of error of the measurement. FT IR measurements before and after catalysis furthermore show no structural changes of the catalyst (Figure S9).

In summary, herein we present a novel material for the room temperature gas phase catalytic hydrogenation of ethene and propene. An Iridium pincer complex was immobilized on a hydroxy‐functionalized microporous polymer network by formation of a metal‐oxo linkage with a conversion efficacy of 75.5 % as determined by ICP‐OES. The successful immobilization of the intact pincer catalyst could be confirmed by solid state ^13^C and ^31^P NMR spectroscopy. X‐ray photoelectron spectroscopy validated the presence of the expected carbon, oxygen, phosphorus and iridium species. A homogeneous distribution of iridium and phosphorus was confirmed by scanning electron microscopy. After immobilization of the molecular catalyst the *SA*_BET_ of the microporous network decreased from 1087 to 451 m^2^ g^−1^ due to the increased specific weight but without loss of the microporous characteristics. The catalytic performance was demonstrated in the room temperature hydrogenation of ethene and propene, which was monitored by ^1^H NMR gas phase spectroscopy. Near‐quantitative conversions were observed after 2 h at room temperature (94 % for ethene and 92 % for propene after 0.5 h) and the recyclability of the catalyst was demonstrated. We believe that this new class of support material ensures the formation of single‐site catalytically active species inside a chemically robust and inert polymer, offering ample opportunity for the field of surface organometallic chemistry.

## Conflict of interest

The authors declare no conflict of interest.

## Supporting information

As a service to our authors and readers, this journal provides supporting information supplied by the authors. Such materials are peer reviewed and may be re‐organized for online delivery, but are not copy‐edited or typeset. Technical support issues arising from supporting information (other than missing files) should be addressed to the authors.

Supplementary
