# Supplementary material for: Immobilization of an Iridium Pincer Complex in a Microporous Polymer for Application in Room‐Temperature Gas Phase Catalysis
Source: Angew Chem Int Ed Engl. 2020 Aug 31;59(45):19830–4. doi: 10.1002/anie.202004092 (PMC7692909; doi:10.1002/anie.202004092)
Supplement: Supplementary file 1 — Supplementary [file ANIE-59-19830-s001.pdf]

Angewandte Chemie
Eine Zeitschrift der Gesellschaft Deutscher Chemiker

Supporting Information

Immobilization of an Iridium Pincer Complex in a Microporous Polymer for Application in Room-Temperature Gas Phase Catalysis

*Michaela König, Massimo Rigo, Nicolas Chaoui, Trung Tran Ngoc, Jan Dirk Epping, Johannes Schmidt, Pradip Pachfule, Meng-Yang Ye, Matthias Trunk, Johannes F. Teichert, Matthias Drieß, and Arne Thomas**

anie_202004092_sm_miscellaneous_information.pdf

SUPPORTING INFORMATION

Table of Contents

Experimental Procedures 2
General 2
Syntheses 3
Catalytic hydrogenation of alkenes 5
Kinetic profiling of catalytic hydrogenation of alkenes 6
Single crystal XRD 6
MAS-NMR spectroscopy 8
Thermogravimetric analysis 9
Physisorption measurements 10
FT IR spectroscopy 11
References 12
Author Contributions 12

Experimental Procedures

General

All inert reactions and manipulations were carried out in an argon atmosphere using standard Schlenk techniques or in an MBraun type MB 120 BG inert atmosphere drybox containing an atmosphere of argon. Benzene and pentane were dried by standard methods and freshly distilled prior to use.

Materials

All chemicals were used as received unless otherwise noted. Anhydrous tetrahydrofuran (99.9 %), anhydrous dichloromethane (99.9 %), n-butyllithium (2.5 M in hexane), 1,4-dibromobenzene (98 %), 1,5-cyclooctadiene (99 %), ethyl 4-bromobenzoate (98 %), boron tribromide (1 M in dichloromethane), diethyl ether (>99.8 %), methyl iodide (>99.0 %) and 4-tritylphenol (97 %) were purchased from Sigma-Aldrich. 2,2‘-bipyridine (99 %) and phenol (>99 %) was purchased from Alfa Aesar. Bis(1,5-cyclooctadiene)nickel(0) (98 %) was purchased from ABCR. Anhydrous dimethylformamide (>99.8 %), sodium hydroxide and tetrahydrofuran (>99.5 %) were purchased from Acros Organics. Acetone (>99.9 %), cyclohexane (>99.5 %), ethyl acetate (>99.5 %), methanol (>99 %) and sulfuric acid (98 %) were purchased from Carl Roth. Anhydrous diethyl ether (99.5 %) and pentane (>99 %) were purchased from Fisher Scientific. 2,6-bis(di-tert-butylphosphinoxy)phenylchlorohydroiridium(III) (>98 %) was purchased from TCI. NaOtBu (98%) was purchased by Acros Organics. Ethene (99.9 Vol%) and Hydrogen (99.999 Vol%) was purchased from Air Liquide. C_6D_6 (99.5 %) was purchased by Deutero.

NMR measurements

^{1}H NMR, ^{13}C NMR, and ^{31}P NMR were recorded on a Bruker Avance II 200 and Bruker Avance 400 MHz spectrometer in the given solvent.
^{11}B MAS, ^{13}C{^{1}H} CP/MAS and ^{31}P MAS measurements were carried out using a Bruker range Avance 400 MHz Solid State spectrometer operating at 128.3 MHz for ^{11}B, 100.6 MHz for ^{13}C, 161.9 MHz for ^{31}P and a Bruker 4 mm double resonance probe-head operating at a spinning rate of 10 kHz.

Physisorption measurements

Argon sorption analyses were conducted at 87 K using an Autosorb-iQ-MP from Quantachrome. The pore size distributions were calculated from the adsorption isotherms by quenched solid density functional theory (QSDFT) using the slit pore model for carbon adsorbents. Before analysis, samples were degassed at 80 °C for 12 h. BET surface areas were determined over a 0.05-0.1 p/p_0 range.

Thermogravimetric analysis

TGA measurements were carried out under air on a Mettler Toledo TGA 1 Stare thermal instrument with a heating rate of 5 K min^{-1}.

ICP/OES

The iridium and nickel contents of the sample were determined by inductively coupled plasma optical emission spectroscopy (ICP-OES) carried out on a Varian ICP-OES 715 ES spectrometer. Prior to the measurement 4 mL of a mixture of conc. sulphuric acid, conc. nitric acid and conc. hydrochloric acid (2:2:1) was added to 5-10 mg of the sample with subsequent microwave treatment at 150 °C for 1 h. The mixture was diluted with distilled water to 50 mL. Standard solutions containing 0.2, 0.5, 1, 2 and 3 ppm nickel and 5, 10, 15, 20 and 25 ppm iridium were used for calibration.

X-ray photoelectron spectroscopy

X-Ray photoelectron spectra were measured on a K-Alpha™ + X-ray Photoelectron Spectrometer System (Thermo Scientific) with Hemispheric 180 ° dual-focus analyzer with 128-channel detector. The X-ray monochromator used micro focused Al-Kα radiation.

FT IR spectroscopy

All manipulations and measurements were carried out in a dry and oxygen-free argon filled glove box. The samples were diluted with FT IR grade KBr in a ratio of 1:20, grinded and pressed into pellets with a hydraulic press at 1.8 tons. FT IR spectra (4000-400 cm^{-1}) were recorded on a Bruker ALPHA FT-IR spectrometer at a resolution of 2 cm^{-1} by accumulating 64 scans. Every sample was measured three times to check reproducibility and spectrometer stability. Blank measurements were conducted before and after each sample. The spectra were acquired in absorbance, baseline corrected and normalized to the band at 3539 cm^{-1}.

Single crystal XRD

Single crystals of the model compound and monomers were grown using a suitable solvent systems, at room temperature. Prior to the analyses, single crystals were immersed in perfluoropolyether as the protecting oil, and fixed on a glass capillary for single-crystal X-ray structure determination. Using these crystal mounted on glass capillary, the crystal-structure determination was performed with an Oxford Diffraction XCalibur (4-Circle Diffractometer , CCD Detector), with Cu K_α (λ = 1.5406 Å) radiation, a Quazar MX multilayer optics monochromator and an Oxford Cryosystems Cryostream 700 plus low-temperature device (T= 150 K). Full-sphere data collection was used with ω and ϕ scans. The data collection and reduction were performed with CrysAlisPro 1.171.38.46 and Olex-2, respectively. The crystal-structure solution was achieved by direct methods as implemented in ShelXT (Intrinsic Phasing),[1] and the structure was visualized with the program Olex 2.[2] The missing atoms were subsequently located from difference Fourier method and added to the atom list. All non-hydrogen atoms were refined with anisotropic displacement parameters.
The ORTEP (Oak Ridge Thermal-Ellipsoid Plot Program)[3] diagrams for the all the structures were plotted with probability level of 50%.

Syntheses

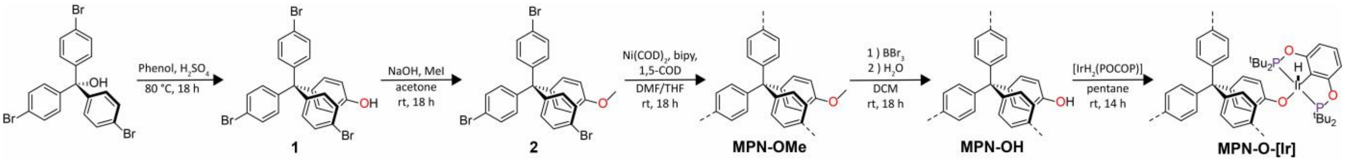

Scheme S1. Syntheses of the methoxytetraphenylmethane polymer network **MPN-OMe** and post-synthetic modification towards hydroxyl functionalized **MPN-OH**.

Syntheses of Monomers

Synthesis of Tris(4-bromophenyl)methanol[4]
1,4-Dibromobenzene (9.46 g, 40.1 mmol, 3.00 eq) was added to a Schlenk flask under argon atmosphere and dissolved in anhydrous THF/Et_2O (50 mL each). The mixture was cooled to -78 °C, and *n*-butyllithium (16 mL of 2.5 M in hexane, 40.0 mmol) was added dropwise during 30 min. After stirring for 1 h at -78 °C ethyl 4-bromobenzoate (2.2 mL, 13.3 mmol, 1.00 eq) was added dropwise to the mixture. The mixture was stirred for 1 h at -78 °C, then allowed to warm to 0 °C and stirred again for 1 h. To the yellowish solution, water (50 mL) and aqueous hydrogen chloride solution (1 M, 50 mL) were added and the aqueous phase was extracted with Et_2O (3 x 30 mL). The organic phase was washed with water and brine and dried over $MgSO_4$. The solvent was removed under reduced pressure to yield the crude product as a yellowish oil which was adsorbed onto silica gel and purified *via* column chromatography from cyclohexane/ethyl acetate (95:5). The obtained solid was recrystallized from ethanol yielding the product as a white solid (4.83 g, 9.77 mmol, 73 %).
^{1}H NMR (200 MHz, $CDCl_3$): δ = 7.45 (d, *J* = 8.8 Hz, 6H), 7.12 (d, *J* = 8.7 Hz, 6H) ppm
^{13}C{H} NMR (50 MHz, $CDCl_3$): δ = 145.1, 131.5, 129.6, 122.1, 81.3 ppm

Synthesis of 4-hydroxyphenyl-tris(4-bromophenyl)methane **1**[5]
Tris(4-bromophenyl)methanol (4.85 g, 9.77 mmol, 1.00 eq) was dissolved in phenol (9.19 g, 97.7 mmol, 10 eq) at 80 °C. The melted phenol served as solvent and no other solvent was added. Sulfuric acid (10 drops, 98 %) was added and the colorless solution turned brown and the mixture was stirred for 18 h at 80 °C. After cooling down to room temperature, an aqueous solution of NaOH (9.1 wt. %, 60 mL) was added while a formation of a white precipitate was observed. The precipitate was filtered and washed with water. The crude product was dissolved in ethyl acetate, adsorbed onto silica gel and purified *via* column chromatography from cyclohexane/ethyl acetate (95:5). The obtained solid was recrystallized from ethanol yielding the product as a white solid (4.59 g, 8.01 mmol, 82 %).
^{1}H NMR (500MHz, $CDCl_3$): δ = 7.38 (d, *J* = 8.7 Hz, 6H), 7.02 (d, *J* = 8.7 Hz, 6H), 6.97 (d, *J* = 8.8 Hz, 2H), 6.72 (d, *J* = 8.8 Hz, 2H) ppm
^{13}C{H} NMR (125 MHz, $CDCl_3$): δ = 154.2, 145.3, 137.7, 132.6, 132.2, 131.0, 120.7, 114.9, 63.4 ppm

Synthesis of 4-methoxyphenyl-tris(4-bromophenyl)methane **2**
1 (2.00 g, 3.50 mmol, 1.00 eq) and NaOH (211 mg, 5.29 mmol, 1.51 eq) were added to acetone (20 mL) and the mixture stirred for 10 min at room temperature. To the clear solution methyl iodide (0.26 mL, 4.18 mmol, 1.20 eq) was added dropwise and the mixture was stirred for 24 h at room temperature, resulting in a suspension. The white precipitate was filtered, dissolved in ethyl acetate, adsorbed onto silica gel and purified *via* column chromatography from cyclohexane/ethyl acetate (95:5). The obtained solid was recrystallized from ethanol yielding the product as a white solid (1.42 g, 2.42 mmol, 69 %).
^{1}H NMR (500 MHz, $CDCl_3$): δ = 7.38 (d, *J* = 8.7 Hz, 6H), 7.05-6.99 (m, 8H), 6.79 (d, *J* = 8.9 Hz, 2H), 3.79 (s, 3H) ppm
^{13}C{H} NMR (125 MHz, $CDCl_3$): δ = 158.1, 145.4, 137.5, 132.7, 132.0, 131.0, 120.6, 113.4, 63.5, 55.4 ppm

Syntheses of Polymers

Synthesis of Methoxytetraphenylmethane Polymer **MPN-OMe**[6]
The polymerization was performed according to standard room temperature Yamamoto reaction procedure. Inside the glovebox, bis(1,5-cyclooctadiene)nickel(0) (1642 mg, 5.97 mmol, 3.50 eq), 2,2'- bipyridine (933 mg, 5.97 mmol, 3.50 eq), and 1,5-cyclooctadiene (0.73 mL, 5.97 mmol, 3.50 eq) were dissolved in anhydrous DMF/THF (160 mL each) and stirred for 15 min while the color of the mixture turned into deep purple. 4-(tris(4-bromophenyl)methyl)anisol **3** (1001 mg, 1.71 mmol, 1.00 eq) was added and the mixture stirred at room temperature for 18 h. The mixture was cooled to 0 °C, aqueous hydrogen chloride solution (10 %, 60 mL) was added dropwise and the mixture stirred at 0 °C for 1 h. The mixture was allowed to warm to room temperature, and stirred for 30 min upon which it turned light blue with aprecipitate. The mixture was filtered off and the colorless precipitate washed with abundant amounts of water, THF and methanol. The product was purified *via* Soxhlet extraction from methanol overnight and dried at 80 °C in vacuum for 6 h to yield **MPN-OMe** as a beige powder. Yield, 595 mg (1.71 mmol repeating units, 100 %).
$^{13}C\{^1H\}$ CP/MAS-NMR (100 MHz): δ = 155, 143, 135, 128, 122, 109, 60, 50 ppm

Synthesis of Hydroxytetraphenylmethane Polymer **MPN-OH**[7]
Methoxytetraphenylmethane polymer **MPN-OMe** (479 mg, 1.38 mmol, 1.00 eq) and anhydrous DCM (50 mL) were added to a Schlenk finger under argon atmosphere. After dropwise addition of BBr_3 solution (1M in DCM, 6.90 mL, 6.90 mmol, 5.00 eq) the mixture was stirred at room temperature for 18 h while the color of the mixture turned into green. Strong gas evolution was observed during dropwise addition of water (50 mL). The mixture was stirred at room temperature for 3 h, filtered off and the off-white precipitate washed with abundant amounts of water, THF and methanol. The product was purified *via* Soxhlet extraction from methanol overnight and dried at 80 °C in vacuum for 6 h to yield **MPN-OH** as off-white, glassy particles. Yield, 430 mg (1.29 mmol repeating units, 94 %).
$^{13}C\{^1H\}$ CP/MAS-NMR (100 MHz): δ = 151, 143, 135, 128, 123, 111, 61 ppm

Synthesis of Reference Hydroxytetraphenylmethane Polymer **MPN-OH** *via* direct polymerization of **1**
The polymerization was performed according to standard room temperature Yamamoto reaction procedure. Inside the glovebox, bis(1,5-cyclooctadiene)nickel(0) (505 mg, 1.83 mmol, 3.50 eq), 2,2'- bipyridine (286 mg, 1.83 mmol, 3.50 eq), and 1,5-cyclooctadiene (0.22 mL, 1.83 mmol, 3.50 eq) were dissolved in anhydrous DMF/THF (50 mL each) and stirred for 15 min while the color of the mixture turned into deep purple. **1** (302 mg, 0.53 mmol, 1.00 eq) was added and the mixture stirred at room temperature for 18 h. The mixture was cooled to 0 °C, aqueous hydrogen chloride solution (10 %, 30 mL) was added dropwise and the mixture stirred at 0 °C for 1 h. The mixture was allowed to warm to room temperature, and stirred for 30 min upon which it turned light blue with a white precipitate. The mixture was filtered off and the colorless precipitate washed with abundant amounts of water, THF and methanol. The product was purified *via* Soxhlet extraction from methanol overnight and dried at 80 °C in vacuum for 6 h to yield a beige powder. Yield, 114 mg (0.34 mmol repeating units, 65 %).
$^{13}C\{^1H\}$ CP/MAS-NMR (100 MHz): δ = 151, 143, 135, 127, 123, 111, 61 ppm

Synthesis of Iridium Pincer Complex **[IrH$_2$(POCOP)]**[8]
(p-HPCP)IrHCl complex (480 mg, 0.76 mmol, 1 eq) and NaO*t*Bu (81 mg, 0.84 mmol, 1.1 eq) were dissolved in nitrogen-free benzene in a Schlenk flask. The mixture was stirred for 1.5 h at room temperature whilst bubbling H_2 through the solution. The reaction mixture was then cooled to 0 °C, and the (frozen) solvent was removed in vacuum for 3 h. The residue was dissolved in pentane and the solution was filtered *via* cannula. The solvent was removed *in vacuo* and the residue dried thoroughly to yield the desired complex as a brown-red powder, which was used without further purification.
^1H NMR (500 MHz, C_6D_6): δ = 7.06 (m, 1H), 6.95 (m, 2H), 1.28 (t, $J_{P\text{-}H}$ = 7.0 Hz, 36H), -17.02 (t, $^2J_{P\text{-}H}$ = 8.3 Hz, 2H) ppm
$^{13}C\{H\}$ NMR (125 MHz, C_6D_6): δ = 170.6 (t, $J_{P\text{-}C}$ = 7.2 Hz), 155.1 (t, $^2J_{P\text{-}C}$ = 6.1 Hz), 131.6 (s), 104.0 (t, $J_{P\text{-}C}$ = 5.6 Hz), 40.2 (vt, $J_{P\text{-}C}$ = 11.7 Hz), 28.8 (vt, $J_{P\text{-}C}$ = 3.5 Hz) ppm
$^{31}P\{H\}$ NMR (202 MHz, C_6D_6): δ = 204.2 (s) ppm

Synthesis of the model compound Tritylphenolate Pincer Complex
To 100mg of the (p-HPCP)IrHH complex and 1eq 4-tritylphenol were added 2 mL of dry and degassed benzene and the solution was stirred. After 2 h the volatiles were removed and the residue was washed with 2 mL of pentane (2 times). The residue was recrystallized from the smallest possible amount of warm toluene, yielding a mixture of crystals of tritylphenol and the product, which were not separated for full characterization.

Immobilization of Iridium Pincer Complex on Polymer for synthesizing **MPN-O-[Ir]**
A solution of the metal precursor in pentane (1.2 eq. circa, 40 mL) was introduced in a 100 mL Schlenk finger containing **MPN-OH** (200 mg, 0.6 mmol OH groups, 1 eq.). An immediate color change was observed in the insoluble polymer from beige to dark red. The resulting mixture was stirred at room temperature overnight (14 h). During the process the solid´s color became lighter due to swelling. The suspension was filtered *via* cannula and the residue washed twice with pentane (5 mL) to remove the excess of metal precursor. Drying in vacuum overnight (14 h) yielded the product as a red powder.
$^{13}C\{^1H\}$ CP/MAS-NMR (100 MHz): δ = 167, 164, 151, 143, 135, 128, 123, 111, 101, 60, 39, 35, 24 ppm
$^{31}P\{^1H\}$ CP/MAS-NMR (162 MHz): δ = 169 ppm

Catalytic hydrogenation of alkenes

In an Ar filled glove box, catalyst **MPN-O-[Ir]** (8 mg) was introduced into a 55 mL- Schlenk flask. The flask was evacuated and filled with a 1:1 mixture of ethene and H_2 to a total pressure of 2 bar at room temperature. After the intended time a gaseous mixture was transferred into a pre-evacuated intermediate pressure NMR tube. The ^{1}H gas phase NMR spectra were measured on a Bruker AvanceIII 500 MHz spectrometer using 16 scans per sample (acquisition time 3.28 s per scan). Due to the absence of a deuterated solvent the samples were recorded without locking and shimming, but using shim settings manually determined on a comparable sample before the measurement. The spectra were analyzed by integration and normalization of the signals for the alkene and corresponding alkane. For the recycling experiments the Schlenk flask was evacuated for 15 min and refilled with a 1:1 mixture of ethene and H_2 to a total pressure of 2 bar at room temperature. The previous described procedure was repeated three times. The mole of ethene was calculated by the ideal gas law and the mole of ethane was determined using the mole fraction. The TON and TOF were calculated based on the catalyst loading determined by ICP (15.7 wt% Ir).
To make sure that relaxation delays were sufficient for quantification relaxation times of pure gaseous ethane and ethene were determined in separate experiments prior to the measurements of the hydrogenation experiments (d_1(ethane) = 754.18 ms, d_1(ethene) = 1.86 s).

Table S1. Collection of different complexes immobilized on various supports applied for gas phase hydrogenation of alkenes.

Entry	Complex	Support	Hydrogenated alkene	Reactor	TOF / min^{-1}	Reference
1	$Rh(C_2H_4)_2$(acac)	Zeolite HY	C_2H_4	Flow[a]	4.4	[9]
2	$Rh(C_2H_4)_2$(acac)	Zeolite NaY	C_2H_4	Flow[a]	1.4	[9]
3	$Rh(C_2H_4)_2$(acac)	MgO	C_2H_4	Flow[b]	1.5	[9]
4	mononuclear Au(III) complex	MgO	C_2H_4	Flow[c]	0.2	[10]
5	$Rh(C_2H_4)_2$[d]	UiO-67	C_2H_4	Flow[e]	2.2	[11]
6	$Rh(C_2H_4)_2$[d]	DAY zeolite	C_2H_4	Flow[e]	6.0	[11]
7	$Rh(C_2H_4)_2$[d]	MgO	C_2H_4	Flow[e]	0.4	[11]
8	$Ir(C_2H_4)_2$[d]	UiO-67	C_2H_4	Flow[e]	0.7	[11]
9	[IrH_2(POCOP)]	NU-1000	C_2H_4	Flow[f]	8.1	[12]
10	[IrH_2(POCOP)][g]	SBA-15	C_2H_4, C_3H_6	Batch[h]	C_2H_4 7.3; C_3H_6 5.1	[13]
11	Rh(COD)	POP	C_3H_6	Flow[i]	0.4	[14]
12	[IrH_2(POCOP)][j]	MPN	C_2H_4, C_3H_6	Batch[k]	C_2H_4 1.9	This work

[a] Feed composition (H_2/C_2H_4/He partial pressures, mmbar) = 142/428/428; 298 K; 1 bar
[b] Feed composition (H_2/C_2H_4/He partial pressures, mmbar) = 100/400/500; 298 K; 1 bar
[c] Feed composition 760 Torr (280 Torr of H_2,40 Torr of C_2H_4, and the balance He), and the total feed flow rate was 100 mL (NTP) min^{-1}; 353 K
[d] Rhodium loadings on UiO-67, DAY zeolite, and MgO were 2.5, 1, and 0.4 wt %, respectively; the iridium loading on UiO-67 was 5 wt %.
[e] TOFs determined from C_2H_4 conversions <5%; catalyst mass = 10−30 mg; feed partial pressures = 50 mbar C_2H_4, 50 mbar H_2 and 900 mbar He; total flow rate = 100 mL (NTP)/min; 298 K.
[f] 296 K; 0.5 bar (rel.); TOF values were determined at conversions below 5 %; ethene : hydrogen ratio of 1 : 1 and making use of 3% C_2H_4 in argon and 3% hydrogen in argon at a final concentration of 1.5 % each
[g] Catalyst loading of 6.1 wt %
[h] 20 mg catalyst, 1 : 1 alkene : hydrogen ratio, initial total pressure: 2 bar absolute, reactor volume: 55 mL; 30 min.
[i] 60 min; 50 mg catalyst; reaction mixture was 30 mL/min of 4% H_2/Ar mixture and 15 mL/min 4% propene/Ar; 298 K
[j] Catalyst loading of 15.7 wt%
[k] 7.8 mg catalyst, 1 : 1 alkene : hydrogen ratio, initial total pressure: 2 bar absolute, reactor volume: 55 mL; 30 min.

Kinetic profiling of catalytic hydrogenation of alkenes

In an Ar filled glove box, catalyst **MPN-O-[Ir]** (3 mg) was introduced into an intermediate pressure NMR tube. The tube was evacuated and filled with a 1:1 mixture of alkene and H_2 to a total pressure of 2 bar at room temperature. After the addition the NMR tube was immediately inserted into the Bruker AvanceIII 500 MHz spectrometer and ^{1}H gas phase NMR spectra were collected. The first spectrum was collected after 4 min (ethene) and 5 min (propene) after starting the reaction. Every spectrum was determined by recording a single scan (acquisition time 3.28 s) with an interval of 10 s (360 points) and 30 s (160 points). The reaction was monitored by integrating the signals of alkene and alkane, normalized to the number of protons and then normalized the sum of integrals to one. The mole fraction was calculated from these integrals and shows the progress of the hydrogenation.

Table S2. Catalytic hydrogenation of alkene at room temperature.[a]

time / h	Conversion / %		TON		TOF / min^{-1}	
	C_2H_4	C_3H_6	C_2H_4	C_3H_6	C_2H_4	C_3H_6
0.1	81	74	31	28	5.2	4.7
0.5	94	92	36	35	1.2	1.2
1	97	94	37	36	0.6	0.6
2	99	98	38	37	0.3	0.3

[a]Reaction conditions: 3 mg catalyst **MPN-O-[Ir]**, no solvent, room temperature, 1:1 ratio of alkene to hydrogen, initial total pressure of 2 bar, reactor volume 2.5 mL. The conversion was determined by integration of the ^{1}H NMR gas phase spectra.

Single crystal XRD

Crystal structure of tetrakis(4-bromophenyl)methane

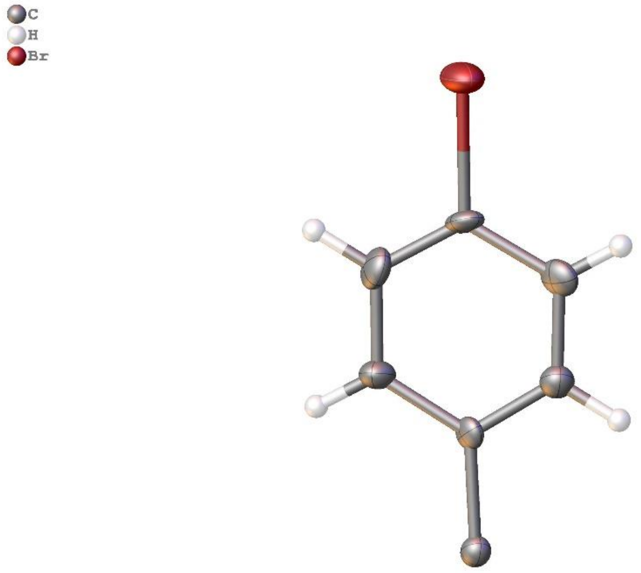

Figure S1. Oak Ridge Thermal-Ellipsoid Plot (ORTEP) diagram (analysis by single crystal X-ray diffraction, 50% probability level) of tetrakis(4-bromophenyl)methane).

Crystal structure of 4-hydroxyphenyl-tris(4-bromophenyl)methane **1**

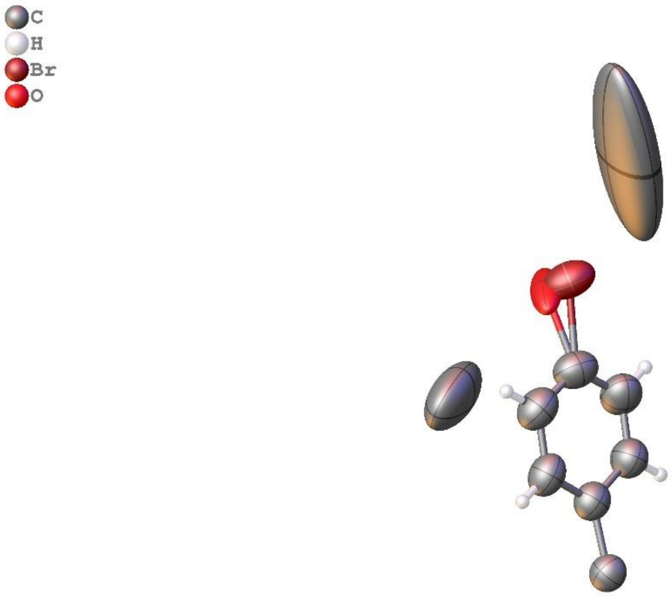

Figure S2. ORTEP diagram (analysis by single crystal X-ray diffraction, 50% probability level) of 4-hydroxyphenyl-tris(4-bromophenyl)methane. Due to the symmetrical structure, the terminal O and Br atoms are disordered (Chemical occupancy: O = 25% and Br = 75%).

Crystal structure of 4-methoxyphenyl-tris(4-bromophenyl)methane **2**

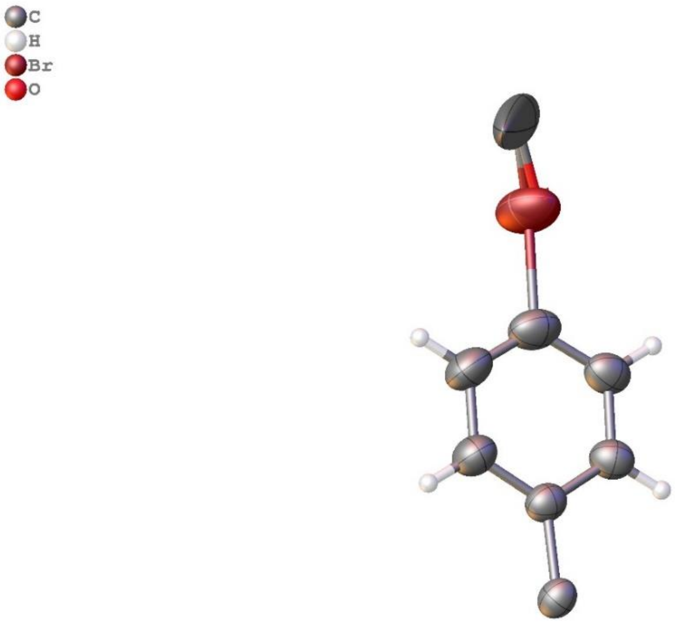

Figure S3. ORTEP diagram (analysis by single crystal X-ray diffraction, 50% probability level) of 4-methoxyphenyl-tris(4-bromophenyl)methane. Due to the symmetrical structure, the terminal O, C and Br atoms are disordered (Chemical occupancy: O = 25%, C = 25% and Br = 75%).

Crystal structure of model compound Tritylphenolate Pincer complex

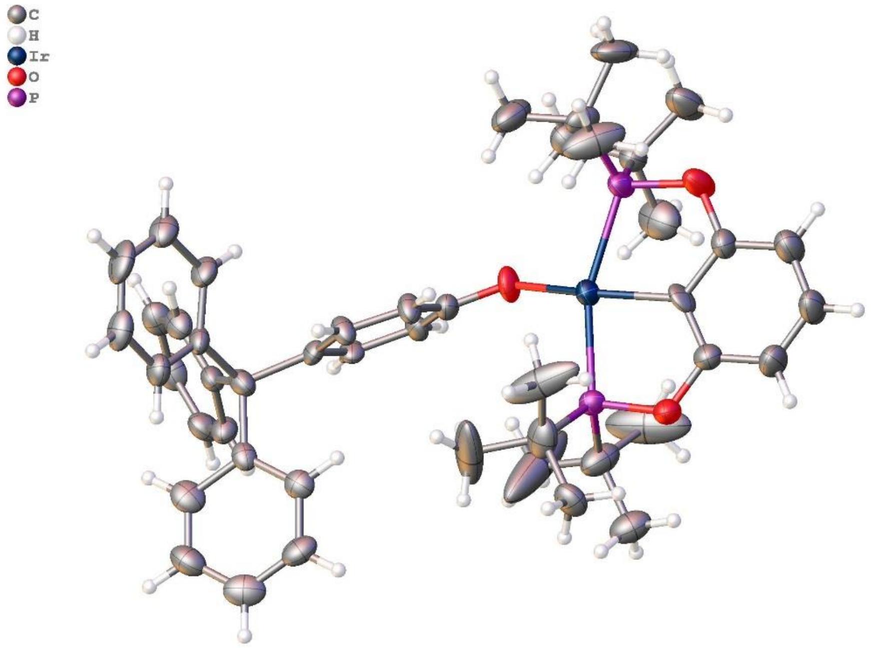

Figure S4. ORTEP diagram (analysis by single crystal X-ray diffraction, 50% probability level) of model compound that mimics the polymer-pincer complex.

MAS-NMR spectroscopy

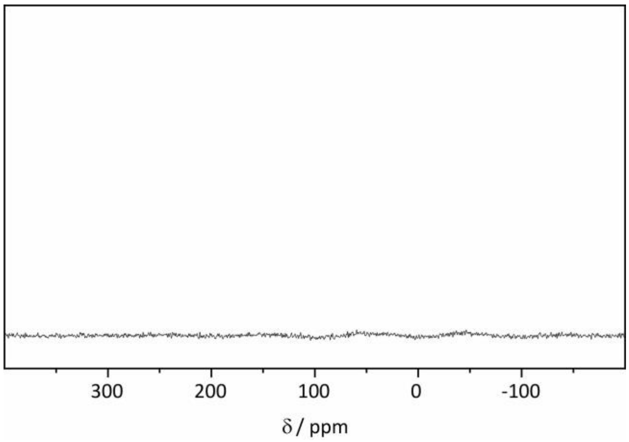

Figure S5. ^{11}B MAS-NMR spectroscopy of **MPN-OH**.

Thermogravimetric analysis

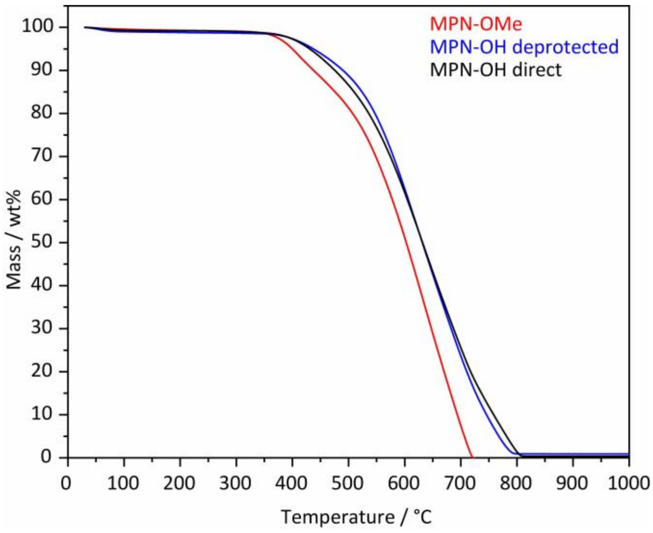

Figure S6. Thermogravimetric analysis of **MPN-OMe** (red), **MPN-OH** *via* demethylation (blue) and **MPN-OH** *via* direct synthesis (black) under air with 5 K min^{-1}.

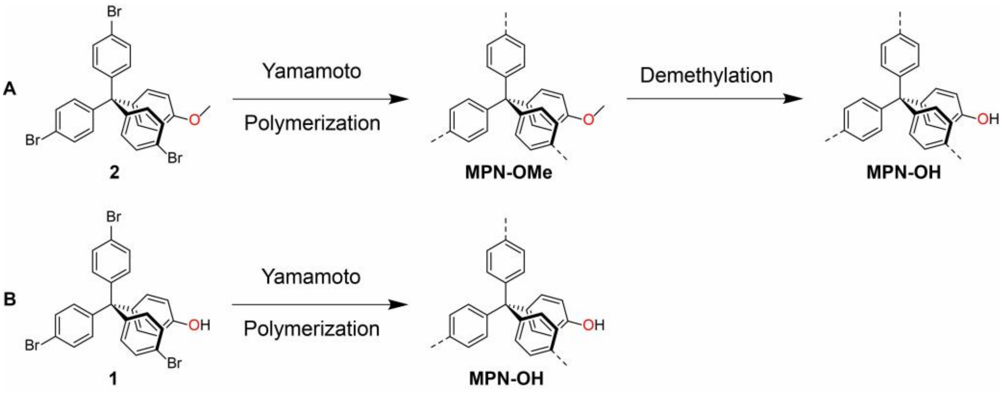

Scheme S2. Syntheses of hydroxyl-polymers *via* **A** demethylation route and **B** direct polymerization of **1.**

Physisorption measurements

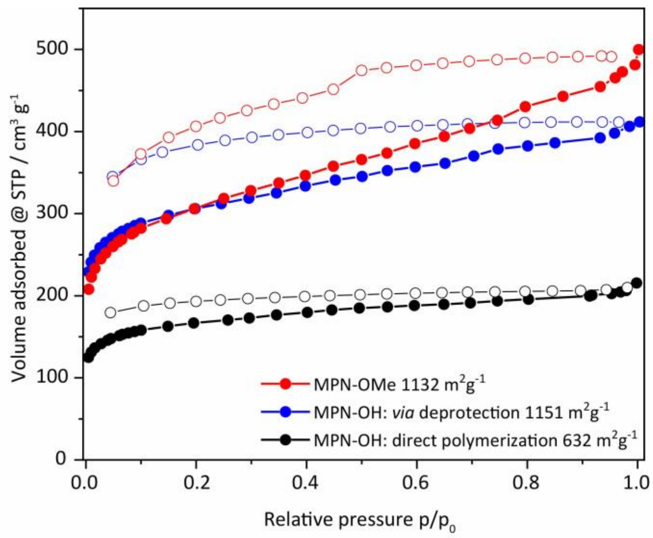

Figure S7. N_2 sorption measured at 77 K for **MPN-OMe** (red), **MPN-OH *via* demethylation** (blue) and **MPN-OH *via* direct synthesis** (black). Note that the SA_{BET} values stated in the manuscript are based on Ar sorption measurements at 87 K shown in **Figure 3** which compare well to the here shown SAs_{BET} from N_2 sorption measurements.

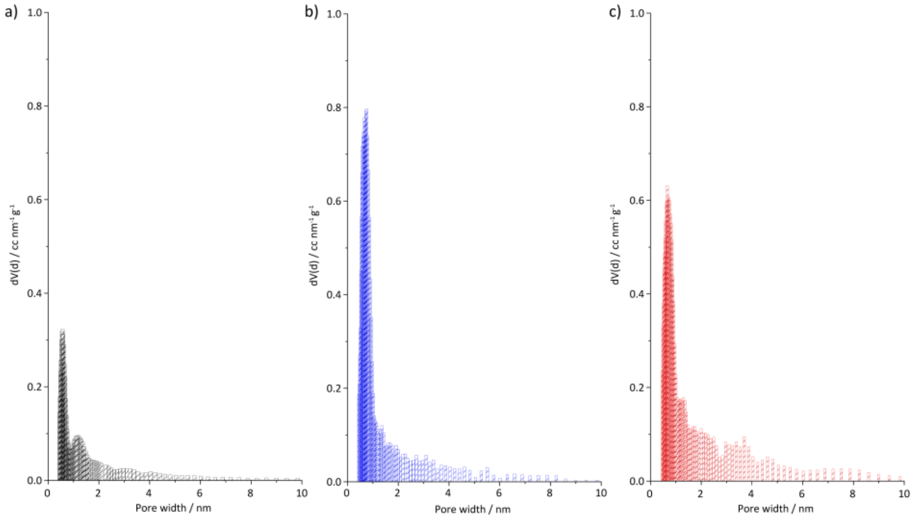

Figure S8. Pore size distribution profile of for a) **MPN-O-[Ir]**, b) **MPN-OH** and c) **MPN-OMe**.

FT IR spectroscopy

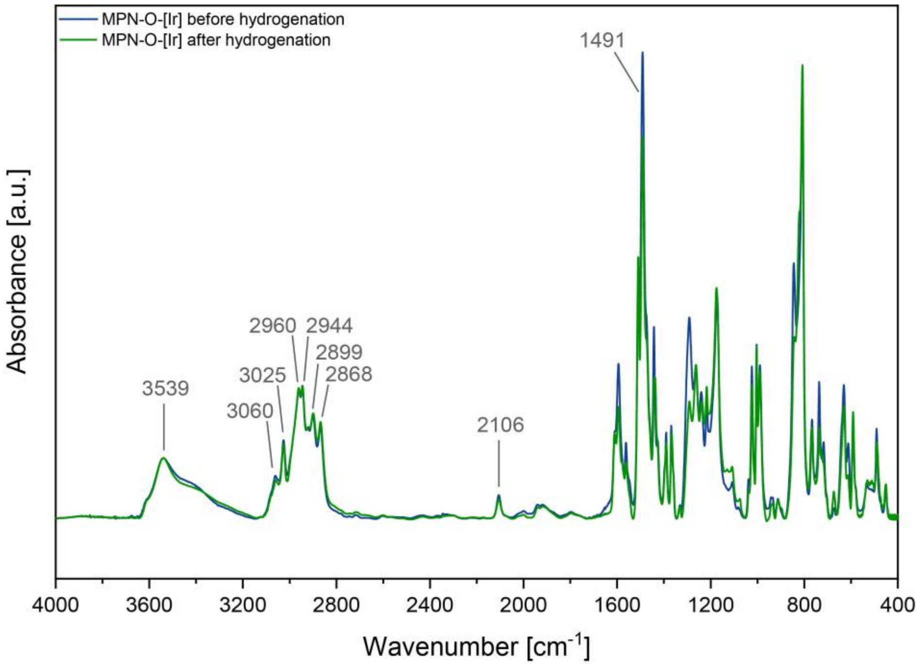

Figure S9. FT IR spectra of **MPN-O-[Ir]** before (blue) and after (green) hydrogenation of ethene. The baseline was corrected, and the intensity was normalized to the band at 3539 cm^{-1}.

A successful immobilization of the iridium pincer complex can be confirmed due to a very similar pattern in the range of 2960 to 2868 cm^{-1} compared to the literature.[13] The intense band at 1491 cm^{-1} can be assigned to the C—H bending of the *tert*-butyl groups of the pincer ligand. The spectra further show a weak band at 2106 cm^{-1}, which we believe to be the Ir—H moiety due to a similar wavelength compared to the literature (2120 cm^{-1}).[13] Remaining phenol moieties could be detected at a broad band between 3650 and 3190 cm^{-1}. This finding is consistent with our suggested functionalization degree of 75 %. Both spectra do not show a significant difference. Therefore, we assume no structural change of the material during the hydrogenation reaction. Also, the active center seems to be intact after the reaction.

References

[1] G. M. Sheldrick, *Acta Crystallogr. Sect. A Found. Crystallogr.* **2008**, *64*, 112–122.

[2] O. V. Dolomanov, L. J. Bourhis, R. J. Gildea, J. A. K. Howard, H. Puschmann, *J. Appl. Crystallogr.* **2009**, *42*, 339–341.

[3] L. J. Farrugia, *J. Appl. Crystallogr.* **2012**, *45*, 849–854.

[4] K. Nikitin, E. Lestini, M. Lazzari, S. Altobello, D. Fitzmaurice, *Langmuir* **2007**, *23*, 12147–12153.

[5] M. E. Gallina, B. Baytekin, C. Schalley, P. Ceroni, *Chem. - A Eur. J.* **2012**, *18*, 1528–1535.

[6] W. Lu, D. Yuan, J. Sculley, D. Zhao, R. Krishna, H.-C. Zhou, *J. Am. Chem. Soc.* **2011**, *133*, 18126–18129.

[7] T. M. Kosak, H. A. Conrad, A. L. Korich, R. L. Lord, *European J. Org. Chem.* **2015**, *2015*, 7460–7467.

[8] I. Göttker-Schnetmann, P. S. White, M. Brookhart, *Organometallics* **2004**, *23*, 1766–1776.

[9] P. Serna, B. C. Gates, *J. Catal.* **2013**, *308*, 201–212.

[10] J. Guzman, *J. Catal.* **2004**, *226*, 111–119.

[11] V. Bernales, D. Yang, J. Yu, G. Gümüşlü, C. J. Cramer, B. C. Gates, L. Gagliardi, *ACS Appl. Mater. Interfaces* **2017**, *9*, 33511–33520.

[12] M. Rimoldi, A. Nakamura, N. A. Vermeulen, J. J. Henkelis, A. K. Blackburn, J. T. Hupp, J. F. Stoddart, O. K. Farha, *Chem. Sci.* **2016**, *7*, 4980–4984.

[13] M. Rimoldi, D. Fodor, J. A. van Bokhoven, A. Mezzetti, *Chem. Commun.* **2013**, *49*, 11314–11316.

[14] S. J. Kraft, G. Zhang, D. Childers, F. Dogan, J. T. Miller, S. T. Nguyen, A. S. Hock, *Organometallics* **2014**, *33*, 2517–2522.

Author Contributions

All authors contributed extensively to the work presented in this paper. M.K. and A.T. conceived the research project. M.K. conducted the synthesis of monomers as well as polymers, and performed the characterizations. M.R. synthesized the pincer complexes and performed the immobilization onto the polymer. M.K. and J.S. designed the hydrogenation experiments and evaluated the hydrogenation of ethene. N.C. and T. T. N. designed and performed the 55 mL hydrogenation experiments. M. K. and T. T. N. performed the recycling experiments for the hydrogenation of ethene and the kinetic profiling of the hydrogenation of propene. M. K. and N. C. analyzed the recyclability of the catalysts for ethene hydrogenation. J.D.E. supported the data collection of the hydrogenation and recycling experiments using NMR techniques. J.S. administrated the project and performed and evaluated the XPS analyses for all the samples. P.P. evaluated the crystal structures for monomers and pincer complex. M.-Y.Y. performed the SEM analyses as well as EDX experiments. M.T. and M.K. developed the polymer synthesis route together. J.F.T. helped and provided the guidance for designing the hydrogenation and recycling experiments. M.D. provided the valuable guidance and helped for immobilizing the pincer complex in the polymer matrix. The whole project was administrated by A.T, with the help from M.K. M.K. and A.T. wrote the manuscript with the input from the other authors.
